# Comparative evaluation of *PD‐L1* expression in cytology imprints, circulating tumour cells and tumour tissue in non‐small cell lung cancer patients

**DOI:** 10.1002/1878-0261.13415

**Published:** 2023-03-23

**Authors:** Mustafa Abdo, Yassine Belloum, David Heigener, Lutz Welker, Sönke von Weihe, Milena Schmidt, Nadine Heuer‐Olewinski, Iris Watermann, Marlen Szewczyk, Jolanthe Kropidlowski, Thais Pereira‐Veiga, Hatice Elmas, Sven Perner, Stefan Steurer, Harriet Wikman, Klaus Pantel, Martin Reck

**Affiliations:** ^1^ LungenClinic Grosshansdorf, Airway Research Center North German Center for Lung Research Grosshansdorf Germany; ^2^ Department of Tumor Biology University Medical Center Hamburg‐Eppendorf Germany; ^3^ Department of Respiratory Medicine Agaplesion Diakonieklinikum Rotenburg Germany; ^4^ Department of Pathology University Medical Center Hamburg‐Eppendorf Germany; ^5^ Institute of Pathology University Hospital Schleswig‐Holstein Lübeck Germany; ^6^ Pathology, Research Center Borstel Leibniz Lung Center Borstel Germany; ^7^ German Center for Lung Research Luebeck and Borstel Germany

**Keywords:** CTCs, cytology imprints, NSCLC, PD‐L1

## Abstract

Alternative sources of tumour information need to be explored in patients with non‐small cell lung cancer (NSCLC). Here, we compared programmed cell death ligand 1 (*PD‐L1*) expression on cytology imprints and circulating tumour cells (CTCs) with PD‐L1 tumour proportion score (TPS) from immunohistochemistry staining of tumour tissue from patients with NSCLC. We evaluated *PD‐L1* expression using a PD‐L1 antibody (28‐8) in representative cytology imprints, and tissue samples from the same tumour. We report good agreement rates on PD‐L1 positivity (TPS ≥ 1%) and high *PD‐L1* expression (TPS ≥ 50%). Considering high *PD‐L1* expression, cytology imprints showed a PPV of 64% and a NPV of 85%. CTCs were detected in 40% of the patients and 80% of them were PD‐L1^+^. Seven patients with *PD‐L1* expression of < 1% in tissue samples or cytology imprints had PD‐L1^+^ CTCs. The addition of *PD‐L1* expression in CTCs to cytology imprints markedly improved the prediction capacity for PD‐L1 positivity. A combined analysis of cytological imprints and CTCs provides information on the tumoural PD‐L1 status in NSCLC patients, which might be used when no tumor tissue is available.

## Graphical abstract

While PD‐L1 assessment via conventional immunohistochemistry is often challenging, cytology imprints and circulating tumour cells (CTCs) appear to be promising alternatives. Here, we compared PD‐L1 expression in matched liquid biopsies and cytology imprints to immunohistochemistry of tissue sections and observed comparable PD‐L1 expression between the different approaches. This further emphasizes cytology imprints and liquid biopsies as complementary approaches to conventional immunohistochemistry.

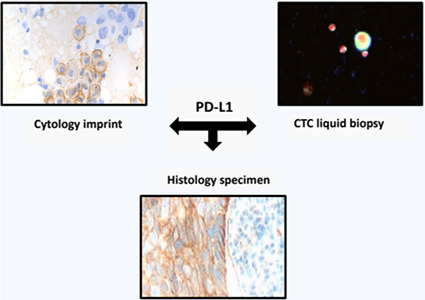

**Abbreviations**
AUC: area under the curve
CI: confidence interval
CTC: circulating tumour cell
EpCAM: epithelial cell adhesion molecule
ICIs: immune checkpoint inhibitors
ISET: isolation by size of epithelial tumour cells
NPV: negative predictive value
NSCLC: non‐small cell lung cancer
PD‐1: programmed cell death protein‐1
PD‐L1: programmed cell death ligand‐1
PPV: positive predictive value
ROC: receiver characteristic operator
ROSE: rapid on‐site evaluation
TPS: tumour proportion score

## 1 Introduction

Lung cancer is the leading cause of cancer deaths worldwide [1]. Non‐small cell lung cancer (NSCLC) contributes to more than 80% of lung cancer diagnosis. Most patients are diagnosed in advanced unresectable disease stages [2]. Immune checkpoint inhibitors (ICIs) targeting programmed cell death protein‐1 (PD‐1) and its receptor ligand‐1 (PD‐L1) have become a mainstay of treatment in NSCLC, particularly, in patients with advanced disease stages who lack druggable molecular alterations [3]. Tumoural *PD‐L1* expression is still the most useful biomarker that predicts treatment response to ICIs [4]. Therefore, *PD‐L1* expression is considered a key factor in selecting NCSLC patients who might benefit from a treatment with ICIs [5]. Based on their use in randomized clinical trials [6, 7, 8], the current standard of care is to quantify tumoural *PD‐L1* expression in histology specimens. Nevertheless, there remains an unmet need to quantify tumoural *PD‐L1* expression in a considerable proportion of NSCLC patients with only cytology samples available for diagnosis [9].

So far, several studies have investigated the feasibility of tumoural *PD‐L1* expression in cytology samples [10]. Many were retrospective analyses or have compared the *PD‐L1* expression between selected paired and matched histology–cytology samples [10]. However, data from prospective real‐world studies elucidating the agreement on *PD‐L1* expression of unpaired histology and cytology samples obtained from the same tumour lesion are still scarce. In addition, the relationship between the detection rate of circulating tumour cells (CTCs) and their *PD‐L1* expression with the tumoural *PD‐L1* expression remains uncertain. While active immune checkpoint receptors represent a potential mechanism of tumour immune evasion [11] and CTCs might be a surrogate marker of tumour immune evasion [12, 13], an association between CTCs detection and tumoural *PD‐L1* expression might exist.

In this prospective study, we sought to investigate the relationship between *PD‐L1* expression on tumour tissue from standard immunohistochemistry with the *PD‐L1* expression of site‐matched cytology imprints of primary tumour lesions and the detection rate of CTCs and their *PD‐L1* expression in patients with NSCLC. This investigation may provide the first evidence of whether alternative sources of tumour cells are informative for the assessment of PDL1 expression.

## 2 Materials and methods

### 2.1 Study design

**Fig. 1:**
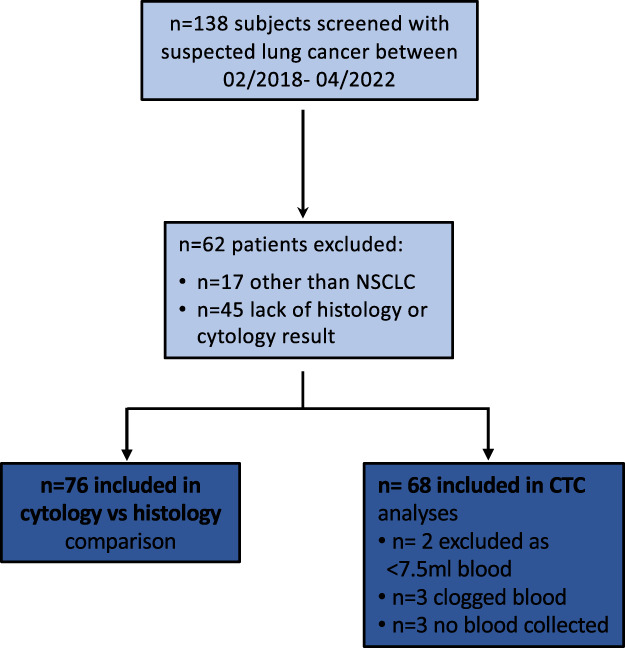
Study cohort recruitment flowchart. A total of 138 patients were screened between 2018 and 2022 of which 76 patients were included in the histology and cytology analysis and 68 in the CTC‐based liquid biopsy analysis.

In this prospective observational single‐centre study, we recruited patients with suspected NSCLC who underwent routine procedures for lung cancer diagnosis at the LungenClinic Grosshansdorf. The analysis included subjects with NSCLC who were 18 ≥ year old. Exclusion criteria were diagnoses other than NSCLC or previous treatment with systemic chemotherapy or immunotherapy (Fig. 1). The written informed consent was obtained before enrolment. The study was approved by the ethics committee at the University of Luebeck (Az. 17‐161) and conducted according to the declarations of Helsinki. Primary tumour specimens were collected via fibreoptic bronchoscopy; this comprises biopsies from endobronchial visible tumour, tumour mucosal infiltration or transbronchial biopsies. Further tumour specimens were obtained from surgical tumour tissue or via ultrasound‐guided percutaneous tumour biopsy. Tumour specimens were smeared in a rapid on‐site evaluation (ROSE), so matched cytology imprints were from the same tumour site. Different pathologists did further PD‐L1 immunostaining on unpaired, yet site‐matched, cytology and histology samples.

### 2.2 Tissue immunohistochemistry and immunocytochemistry

The immunohistochemical staining was performed on 4‐μm‐thick sections obtained from formalin‐fixed paraffin‐embedded tumour tissue. The quantification of *PD‐L1* expression was done by estimating the number of PD‐L1‐positive tumour cells as a percentage of all tumour cells in both histology sections and cytology imprints. Tumour proportion score (TPS) was determined as the percentage of cells, which are PD‐L1 positive to the total number of cells that included at least 100 viable tumour cells [14]. Staining of samples was done with the antibody clone Dako 28‐8 according to standard operating procedures [15]. Although the 28‐8 antibody clone was used in this study as it is the standard antibody used for routine clinical diagnostics of NSCLC at the department of Pathology in Hamburg, a high degree of agreement between the 28‐8 and 22C3 PD‐L1 antibody clones for histological and cytological samples staining results have been described before [10, 16]. A consistently lower positivity rate has been described for the SP142 antibody [17, 18].

### 2.3 Circulating tumour cell‐based liquid biopsy

We used the Parsortix® Technology (ANGLE plc, Guildford, UK) to detect CTC from 7.5 mL blood collected in Transfix tubes (CTC‐TVT tubes, CYTOMARK, Buckingham, UK) as previously described [19, 20]. The Parsortix technology has been extensively evaluated including several studies on NSCLC and also as part of multicentre ring trials (CANCER ID consortium) [21, 22, 23]. Cells enriched by the Parsortix® system were directly harvested into cytospin funnels, centrifuged onto a glass slide (RCF 190 ***g***), dried overnight and stored at −80 °C until further processing. For staining, slides were brought to room temperature and fixed with 0.5% PFA for 10 min. Cells were washed with 0.5 mL of 1× PBS three times for 3 min each. 10% AB‐ serum (BioRad, Rüdigheim, Germany) was applied for blocking (20 min). Unconjugated rabbit anti‐human PD‐L1 antibody, clone HL1041 (GTX635975, 1 : 100) was incubated over night at 4°, after which cells were washed with 0.5 mL of 1× PBS three times for 3 min. BD Horizon™ BV421 goat anti‐ Rabbit (BD Biosciences, San Jose, CA, USA, 1 : 200) was used as a secondary antibody and incubated for 45 min. Following the three additional washing steps, directly eFluor560 conjugated pan‐keratin (AE1/AE3‐eBioscience, San Diego, CA, USA, 1 : 200), PerCP‐labelled CD45 (clone H130‐Miltenyi Biotec, Bergisch Gladbach, Germany, 1 : 200) and DRAQ5™ for nuclear staining (BioLegend, San Diego, CA, USA, 1 : 5000) antibodies were incubated for 60 min. Subsequently, cytospins were covered with Prolong Gold Antifade Reagent (Thermo Fisher Scientific, Dreieich, Germany), sealed with a cover slip and examined by fluorescence microscopy. Keratin‐positive, DRAQ5 (nuclear)‐positive and CD45‐negative cells with intact morphology were defined as tumour cells. H1975 was used as a positive control for *PD‐L1* expression, while MFC7 was used as a negative control.

Rabbit anti‐human PD‐L1 antibody, clone 28.8 was optimized to detect cell surface PD‐L1 in formalin‐fixed paraffin‐embedded human tumour tissue specimens [15] and its specificity was demonstrated by antigen competition and genetic deletion of PD‐L1 in tumour cell lines. It is an approved companion test antibody. However, its use in the immune‐fluorescence setting is poorly investigated as the antibody is mainly used for IHC approaches. Rabbit anti‐human PD‐L1 antibody, clone HL1041 (Genetex, Irvine, CA, USA) targeting PD‐L1 cell membrane as well, was compared to other antibodies frequently used for immunofluorescence PD‐L1 staining, including PD‐L1 E1L3N clone and D8T4X clone (Cell Signalling Technology, San Diego, CA, USA, both). *PD‐L1* expression was assessed using cell lines with known different *PD‐L1* expression levels [12]. Although these clones worked alike, a slightly higher signal was observed for the newly released clone HL1041 and thus this antibody was used for the CTC assays.

### 2.4 Statistical analysis

We used a receiver operator characteristic (ROC) to evaluate the percent*PD‐L1* expression from cytology imprints and the expression of *PD‐L1* in CTCs as predictors for positive *PD‐L1* expression (tumour cells expression score ≥ 1%) and *PD‐L1* high expression (tumour cells expression score ≥ 50%) defined according to standard immunohistochemistry staining. We used Fisher exact test to identify differences in clinical variables between the study groups.

To examine the correlation between two continuous variables, we used Pearson's test. Statistical analyses were performed using r (version 4.2.1, R Foundation, Vienna, Austria). An alpha error of less than 5% was considered statistically significant.

## 3 Results

### 3.1 Study population

One hundred and thirty‐eight subjects with suspected lung cancer were screened. We excluded subjects who had a diagnosis other than NSCLC (*n* = 17) or due to lacking histology specimens or evaluable cytology imprints (*n* = 45) as shown in the flow chart (Fig. 1). The final analysis included 76 patients, of whom the majority had non‐squamous non‐small lung cancer in locally advanced or metastasized disease stages, Table 1. Nearly 80% of the specimens were collected via fibreoptic bronchoscopy; this comprised biopsies from endobronchial visible tumour, tumour mucosal infiltration and transbronchial biopsies. Further tumour specimens were obtained from surgical tumour tissue or via ultrasound‐guided percutaneous tumour biopsy (Table 1).

**Table 1:** Patients' characteristics. PD‐L1 high expression, PD‐L1 tumour cell expression score ≥ 50%; PD‐L1 positivity, PD‐L1 tumour cells expression score ≥ 1%; PD‐L1, programmed cell death‐ligand 1; UICC, Union for International Cancer Control.

Age, years	65.2 ± 9.0
Sex, male *n* (%)	50 (67)
Histological subtypes, *n* (%)	
Adenocarcinoma	40 (52)
Squamous cell carcinoma	29 (38)
Not otherwise specified	7 (10)
UICC staging, *n* (%)	
IA	7 (9.0)
IB	2 (2.6)
IIA	2 (2.6)
IIB	3 (3.9)
IIIA	9 (11.8)
IIIB	15 (19.7)
IIIC	2 (2.6)
IVA	18 (23.6)
IVB	18 (23.6)
Type of biopsy, *n* (%)	
Endoscopic	60 (79)
Surgical	11 (14.5)
Percutaneous needle biopsy	5 (6.5)
PD‐L1 expression score, %	
Histology specimens	36.1 ± 36
Cytology smears	40.4 ± 33
PD‐L1 positivity, *n* (%)	
Histology specimens	67 (88.1)
Cytology smears	67 (88.1)
PD‐L1 high expression, *n* (%)	
Histology specimens	29 (38.1)
Cytology smears	36 (47.3)

### 3.2 *PD‐L1* expression in the tumour tissue samples and in cytological imprints

We found a moderate correlation of percent *PD‐L1* expression between cytology imprints and the matched histology specimens (*R* = 0.58, *P* < 0.001). Likewise, we observed a similar estimation for the number of patients with positive *PD‐L1* expression (TPS ≥ 1%); yet, a higher estimation of patients with high *PD‐L1* expression (TPS ≥ 50%) according to cytology imprints than in histology specimens (Table 1). Compared to percent *PD‐L1* expression from standardized immunohistochemistry, the predictive capacity of cytology imprints of PD‐L1 positivity (≥ 1%) indicated a positive predicted value (PPV) of 91%, a negative predicted value (NPV) of 33%, AUC = 78% [95% CI: 65–90%]. Considering high *PD‐L1* expression (≥ 50%), cytology imprints showed a PPV of 64% and a NPV of 85%, AUC = 79% [95% CI: 67–91%].

The overall agreement on *PD‐L1* positivity for the whole cohort was 84%. Positive agreement on PD‐L1 positivity was seen in 61 cytology imprints out of 67 matched histology specimens; yielding a positive agreement rate of 91.0%. The negative agreement rate was 33.3% and was only seen in three cytology imprints out of nine matched histology specimens. The overall agreement on PD‐L1 high expression was 82.8%. Positive agreement on PD‐L1 high expression was 79%; seen in 23 cytology imprints out of 29 matched histology specimens and the negative agreement was 85%, seen in 40 cytology imprints out of 47 matched histology specimens.

Furthermore, the overall correlation of percent *PD‐L1* expression between cytology imprints and histology specimens was higher in surgical specimens (*R* = 0.67, *P* < 0.01) than in non‐surgical specimens (*R* = 0.56, *P* < 0.01). Moreover, specimens obtained from surgically resected tumour tissue yielded a greater cytology–histology agreement than non‐surgical specimens, that is, those that were obtained via fibreoptic bronchoscopy or percutaneous tumour biopsy. The cytology–histology agreement on *PD‐L1* high expression was 100% versus 75% in surgical versus non‐surgical specimens respectively. Nevertheless, the cytology–histology agreement on PD‐L1 positivity in surgical specimens (91%) was yet comparable to the agreement from non‐surgical specimens (89%).

### 3.3 *PD‐L1* expression in CTCs

Sixty‐eight out of the 76 samples were assessed for *PD‐L1* expression on CTCs. Eight samples were excluded due to clogged or not evaluable blood samples (*n* = 3), low blood volume (< 5 mL, *n* = 2) or missing liquid biopsy samples (*n* = 3). CTCs were detected in 27/68 samples (39.7%). The detection rate of CTCs was comparable between patients with: non‐resectable versus resectable (OR 1.59 [95% CI 0.49–5.14], *P* = 0.43), non‐squamous versus squamous (OR 0.64 [95% CI 0.20–1.94], *P* = 0.45), negative versus positive *PD‐L1* expression (OR 0.86 [95% CI 0.13–6.44], *P* = 1.0) and non‐high versus high *PD‐L1* expression tumours (OR 0.92 [95% CI 0.29–2.78], *P* = 1.0), or M1 versus M0 disease stages (OR 1.18 [95% CI 0.40–3.50], *P* = 0.80). Yet, CTC detection rate showed a tendency to be elevated in patients with stage IVB versus patients with all other disease stages (OR 3.52 [95% CI 0.90–15.5], *P* = 0.063) and was significantly higher in patients with stage IVB than those with stage IVA (OR 5.48 [95% CI 0.98–37.6], *P* = 0.032; Table 2).

**Table 2:** CTC cohort characteristics. CTCs, circulating tumour cells; PD‐L1, programmed cell death‐ligand 1; UICC, Union for International Cancer Control.

Characteristics	*n* (%)	*n* CTC^+^ (%)	*n* PD‐L1^+^ CTC (%)
CTC patients' cohort	68 (100)	27 (39.7, 1–13^a^)	21 (77.8)
Sex			
Male	43 (63.2)	16 (37.2)	12 (57.1)
Female	25 (36.8)	11 (44.0)	9 (42.9)
Histological subtypes			
Adenocarcinoma	36 (52.9)	18 (50.0)	13 (72.2)
Squamous cell carcinoma	27 (39.7)	9 (33.3)	8 (88.9)
Not otherwise specified	5 (7.4)	0	0
UICC staging			
M0	37 (54.4)	14 (37.8)	11 (78.6)^b^
M1	31 (45.6)	13 (41.9)^c^	10 (76.9)^d^

^a^ CTC range in the cohort.

^b^ Include one patient with keratin^+^ and PD‐L1^+^ cluster.

^c^ Include 1 patient with keratin^+^ and PD‐L1^−^ cluster.

^d^ Include two patients with keratin^+^ and PD‐L1^+^ cluster.

**Fig. 2:**
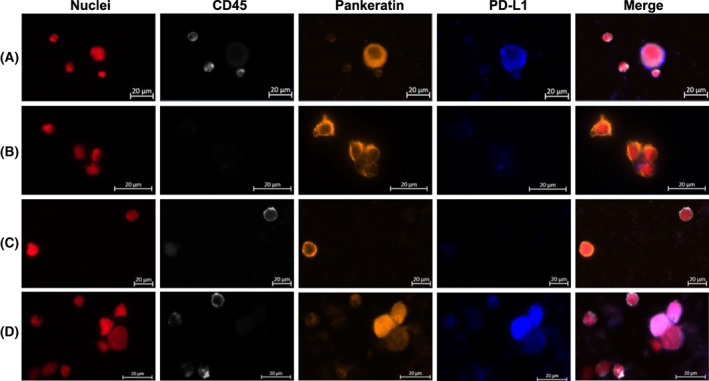
Example of fluorescence microscopy of circulating tumour cells. Programmed cell death ligand‐1 positive (A, D) versus Programmed cell death ligand‐1 negative (B, C) circulating tumour cells. Red: nuclei. White: CD45. Orange: Pankeratin. Blue: Programmed cell death ligand‐1. The scale bar of 20 μm applies to all pictures.

The average CTC number was 2.7 CTCs per 7.5 mL of blood (range 1–13 CTCs; Table 2). PD‐L1^+^ CTCs were detected in 21 blood samples (77.8%) with an average of 1.4 PD‐L1^+^ CTC per blood sample (range 1–6). In these 21 samples, the PD‐L1^+^ CTC subset represented 10.0% to 100.0% of all detected CTCs. One M0 (stage III) patient had PD‐L1^+^ CTC cluster of three CTCs, while two‐stage IV patients had each one CTC cluster with all cells positive for PD‐L1. Only one stage IV patient had a cluster negative for PD‐L1 (Table 2). Examples of single CTC and CTC cluster staining with positive versus negative PD‐L1 expression are presented in Fig. 2.

We assessed for agreement on positive *PD‐L1* expression between histology specimens and CTCs in patients who had at least one CTC (*n* = 27). Here, we found a relatively good overall agreement of 66.7%, with three patients showing PD‐L1^+^ CTCs yet, a negative *PD‐L1* expression in histology specimens (Table 3). When considering high *PD‐L1* expression, the agreement rate dropped to 51.9%. Of patients with high *PD‐L1* expression in histology specimens, 90.0% had PD‐L1^+^ CTCs; however, PD‐L1^+^ CTCs were also detected in 70.6% of patients who had negative *PD‐L1* expression (< 1%) in histology specimen (Table 3).

**Table 3:** Agreement between PD‐L1 expression in tumour tissue biopsy (≥ 1 and ≥ 50% of tumour cells) and CTCs (*n* = 27). CTCs, circulating tumour cells; n.d., non‐determined for absence of events (0); PD‐L1, programmed cell death‐ligand 1.

		PD‐L1 expression in tumour biopsy (≥ 1% of tumour cells)		Positive; negative predictive value
		Yes, *n* (%)	No, *n* (%)	
		24 (88.89)	3 (11.11)	
Presence of PD‐L1^+^ CTCs	Yes, *n* (%)	18 (75.0%)	3 (100.0%)	85.7%; n.d.
	No, *n* (%)	6 (25.0%)	0	

		PD‐L1 expression in tumour biopsy (≥ 50% of tumour cells)		
		Yes, *n* (%)	No, *n* (%)	
		10	17	
Presence of PD‐L1^+^ CTCs	Yes, *n* (%)	9 (90.0%)	12 (70.6%)	42.9%; 83.3%
	No, *n* (%)	1 (10.0%)	5 (29.4%)	

Furthermore, the overall agreement on positive *PD‐L1* expression between cytology imprints and CTCs was 62.9%. Similar to histology specimen, the agreement rate dropped to 51.9% when high *PD‐L1* expression in cytology imprints was considered (Table 4). The addition of CTCs PD‐L1 expression has markedly improved the prediction capacity of cytology imprints for PD‐L1 positivity; AUC = 91% [95% CI: 79–100%] and for high *PD‐L1* expression; AUC = 84% [95% CI: 69–100%] from standardized immunohistochemistry.

**Table 4:** Agreement between PD‐L1 expression in cytological smears (≥ 1% and ≥ 50% of tumour cells) and CTCs (*n* = 27). CTCs, circulating tumour cell; n.d., non‐determined for absence of events (0); PD‐L1, programmed cell death‐ligand 1.

		PD‐L1 expression in cytological smears (≥ 1% of tumour cells)		Positive; negative predictive value
		Yes, *n* (%)	No, *n* (%)	
		23 (85.19)	4 (14.81)	
Presence of PD‐L1^+^ CTCs	Yes, *n* (%)	17 (26.1)	4 (100.0)	80.9%; n.d.
	No, *n* (%)	6 (73.9)	0	

		PD‐L1 expression in cytological smears (≥ 50% of tumour cells)		
		Yes, *n* (%)	No, *n* (%)	
		12 (14.44)	15 (55.56)	
Presence of PD‐L1^+^ CTCs	Yes, *n* (%)	10 (83.3)	11 (73.33)	47.6%; 66.7%
	No, *n* (%)	2 (16.7)	4 (26.66)	

## 4 Discussion

The evaluation of tumoral *PD‐L1* expression is essential for selecting patients with NSCLC who might benefit from treatment with ICIs. So far, the evaluation of tumoral *PD‐L1* expression is only validated for histology specimens [6, 7, 8], excluding a considerable proportion of NSCLC patients for whom no tumour tissue is available [9, 10]. In this prospective study, we therefore compared *PD‐L1* expression from standard immunohistochemistry with the *PD‐L1* expression of cytology imprints and CTCs. Tumour samples were obtained from the primary tumour site through various biopsy procedures and evaluated independently by different pathologists. Using this approach, we sought to avoid sampling bias and to represent real‐world data on the potential use of cytology imprints and CTCs for examining *PD‐L1* expression. Though *PD‐L1* assessment was confirmed as a predictive biomarker for histological samples, the evaluation of *PD‐L1* expression on paired cytological specimen has also shown comparable results [14, 16, 24, 25]. However, to our knowledge, this is the first study comparing *PD‐L1* expression on paired histological, cytological and CTC‐based liquid biopsy specimen.

Overall, our data indicate a good cytology–histology agreement for both PD‐L1 positivity and high expression. Furthermore, our study demonstrates the added role of CTCs‐*PD‐L1* expression as the combination of liquid biopsy and cytology has markedly improved the positive prediction capacity for PD‐L1 positivity and high expression. Noteworthy, cytology imprints yielded excellent positive agreement (91%), yet a poor negative agreement, on PD‐L1 positivity. This might indicate that cytology imprints overestimate PD‐L1 positivity, that is, PD‐L1 tumour cells expression score ≥ 1% in PD‐L1 negative tumours as per immunohistochemistry. However, cytology imprints also yielded a good negative agreement on *PD‐L1* high expression, that is, *PD‐L1* tumour cells expression score ≥ 50% with a NPV of 85%, AUC = 79% [95% CI: 68–91%], indicating good capacity of these imprints in ruling out patients who might not qualify for a first‐line monotherapy with ICIs. Our data also indicate that samples obtained from surgically resected tumour tissue might yield a greater cytology–histology agreement than those obtained via fibreoptic bronchoscopy or percutaneous tumour biopsy.

Our findings regarding the PD‐L1 cytology–histology agreement are in line with the finding of other previous studies that reported an agreement rate of between 65–100% for both PD‐L1 positivity and high *PD‐L1* expression [10]. Many factors contribute to cytology–histology disagreement as well as to the heterogeneity in the reported agreement rates. This includes the intra‐tumour heterogeneity of *PD‐L1* expression [26], the number of tumour cells in cytology samples [27] as well as the discordance due to the applied diagnostic tools in tumour sampling and staining procedures including used antibodies [28]. Further, the type of cytology specimens might have an impact as cytological cell blocks [29, 30, 31] which demonstrated better agreement with histology specimen than cytology imprints [14, 32, 33].

In this study, we also compared *PD‐L1* expression between standard immunohistochemistry and a label‐independent, microfluidic‐based CTC enrichment system. As previously reported, our data confirm that the Parsortix system reliably detects CTCs in liquid biopsies from patients with NSCLC [20, 23]. The detection rate of CTCs in our cohort was nearly 40%. Noteworthy was that CTCs detection rates were rather comparable between patients with resectable versus non‐resectable as well as between patients with metastatic (M1) and non‐metastatic (M0) disease stages. Nevertheless, the subgroup analysis has revealed that CTCs detection rate is significantly higher in patients with stage IVB (64%) than those with stage IVA (23%) or patients with non‐metastasized tumours (37%).

Here, we also report that most CTCs (nearly 80%) showed positive *PD‐L1* expression. *PD‐L1* expression in CTCs of patients with NSCLC has already been assessed using various different enrichment techniques and PD‐L1 antibodies [34]. Kulasinghe et al. [35] used the microfluidic‐based ClearCell FX system to assess for CTC in a smaller cohort of patients with advanced NSCLC and reported a CTCs detection rate of 51% with 65% PD‐L1‐positive cells. In a further study, the same authors detected CTC in 60% of patients with stage IV NSCLC and 56% of selected patients with CTC were PD‐L1 positive [36]. The absence of clinical relevance of *PD‐L1* expression on CTCs prior to therapy has also been reported in Guibert et al. study [37]. The authors used the size‐based separation ISET platform to yield a high CTC positivity of 93% at baseline (*n* = 89/96), with 83% of these patients expressed PD‐L1 on at least one CTC. Sinoquet et al. [38] used the EpCAM‐based CellSearch enrichment method, and reported a 43.4% of CTC positivity (*n* = 54 patients) with a low PD‐L1^+^ CTC rate of 21.7%. Janning et al. [19] reported a 68.5% CTC positivity rate and 81.9% of PD‐L1^+^ CTC in late‐stage NSCLC patients using the same system as ours. Overall, with a CTC positivity of nearly 40% and PD‐L1^+^ CTC of 78%, our data align with what was previously reported in literature. We suggest that the observed variability could be attributed to the different enrichment techniques including the antibody that has been used. Still, the sample size and rather low positivity rate is clearly a limit of our study that could cause a bias in or data.

Furthermore, agreement rates between *PD‐L1* expression on CTCs versus cytological imprints yielded similar results to CTCs versus histology specimen; 62.9% with PD‐L1 ≥ 1% versus 51.9% with high *PD‐L1* expression (≥ 50%). Notable was that the agreement rate between *PD‐L1* expression on CTCs versus tissue was relatively low for almost all the previously described studies. Only Ilie et al. [39] reported a high agreement (93%) of PD‐L1^+^ CTC with matched tissue using the ISET platform. In our study, a higher agreement was observed compared to many other studies. This could be due to the use of a new more sensitive PD‐L1 antibody that was shown to be very sensitive when used for immunofluorescence staining. By using this new staining protocol, a moderate to high agreement of 66.7% was observed when at least 1% of cells expressed *PD‐L1*. However, the agreement drops to 51.9% when increasing the threshold of PD‐L1 tissue positivity to ≥ 50%. Indicating that preferentially the PD‐L1‐positive cells enter or survive in the blood circulation.

For further refinement and in order to increase predictive accuracy, few limitations inherent to our study need to be considered. Though we followed standard procedure and kept the cell quantity as previously advocated (at least 100 viable tumour cells), the sample size might be a limit for which *PD‐L1* expression might be underestimated. Nearly 80% of the specimens were collected via fibreoptic bronchoscopy making the chance of underestimating the PD‐L1 content higher [40]. This limit further highlights the importance of a combined approach for PD‐L1 assessment and suggests that the use of a liquid biopsy approach through CTC analysis might improve PD‐L1 predictive accuracy.

## 5 Conclusions

Our study shows that the tissue biopsy and the consequent smear imprint at a single tumour site or a specific time point is insufficient to represent the overall status of PD‐L1 on tumour tissue. There is obvious spatial and temporal heterogeneity of *PD‐L1* expression on tumour tissue that cannot be unravelled by conventional tissue biopsy and cytological imprints which might explain the low agreement rate when considering a 50% positivity threshold. By assessing *PD‐L1* expression on CTCs in a minimal invasive approach and a real‐time detection using a label‐independent, microfluidic system, may represent a complementary source for PDL1 immunostaining, which also makes the dynamic monitoring of PD‐L1 during treatment more convenient for physicians and less invasive for patients [41]. Still, future larger prospective studies assessing all these biomarkers in NSCLC patients receiving ICI are needed to be performed to assess the sensitivity and specificity of each approach.

## Conflict of interest

The authors declare no conflict of interest.

## Author contributions

MA, YB, HW, KP and MR were involved in study concept and design and drafted the manuscript. MA, YB, HW and MR carried out data analysis. MA, YB, MSc, NH‐O, IW, MSz, JK, TP‐V and HE were involved in patient sample collection. MA, YB, DH, LW, JK, SW and TP‐V performed the experiments. MA, YB, DH, LW, SW, SP, SS, HE and HW carried out data interpretation. All authors have read and agreed to the published version of the manuscript.

## Data Availability

Data are available upon reasonable request form the corresponding author.
